# Does financial inclusion promote investment and affect residents' happiness?—Evidence from China

**DOI:** 10.3389/fpsyg.2022.988312

**Published:** 2022-08-22

**Authors:** Qiuyan Xu, Wu Sun

**Affiliations:** ^1^Department of Economics, School of Economics and Management, Shihezi University, Shihezi, China; ^2^Department of Finance, International Business College, Shandong Technology and Business University, Yantai, China

**Keywords:** financial inclusion, investment, residents' happiness, China, CGSS

## Abstract

With the rapid development of inclusive finance, the popularity of financial services is increasing, and the level of financial literacy of residents has gained. Using data from the years 2013, 2015, and 2017 China General Social Surveys (CGSS) and the China Digital Inclusive Finance Development Index to analyze residents' investment behavior in China, this study finds that inclusive finance significantly increased residents' investment participation and decrease their sense of happiness at the same time. This study demonstrates the effectiveness of China's financial inclusion policy and provides ideas for its further improvement.

## Introduction

With the rapid development of China's economy and the rapid growth of residents' income, the market demand for investment products has become increasingly high. In order to improve the coverage of financial services, China has vigorously developed inclusive finance, which has improved the availability of financial services, unblocked credit channels, and gained an increase in the level of financial literacy among residents. Inclusive finance has not only eased the financing constraints of enterprises, improved the efficiency of capital allocation in the market, but also raised the income of residents.

As the rapid growth of residents' financial literacy and the rapid accumulation of household wealth, more and more households are undertaking appropriate financial investment and household asset allocation to obtain returns and prevent risks. The increase in financial asset allocation will improve the overall level of household returns and diversify investment risks. Therefore, the development of inclusive finance is an important means to increase the proportion of financial assets of Chinese households. According to the Report on Financial Asset Allocation Risks of Chinese Households published by Southwest University of Finance and Economics, the proportion of financial assets of households in the US and Japan reached 68.8 and 61.1% in 2015, while the proportion was only 12.4% in China, which is relatively low (China Household Finance Survey and Research Center, 2016). Financial inclusion will promote investment in risky assets (Liao and Zhou, 2020; Zhou et al., 2020), and enhance the proportion of financial assets.

The term inclusive finance was first formally introduced by the United Nations in 2005 and refers to a financial system that can effectively and comprehensively provide services to groups from all segments of society (Yi and Zhou, 2018). Its business types mainly include inclusive microfinance business, consumer finance, rural revitalization, poverty alleviation, and so on. In recent years, the development of innovative technologies such as information technology, big data, and cloud computing has driven the progress of digital finance. Since the implementation of the financial inclusion development strategy in 2011, financial inclusion has rapid development, and the total digital financial inclusion index of all provinces increased significantly, with an average annual growth rate of 37.90% (Guo H. et al., 2020). The average annual development rate of inclusive finance reached 66% between 2011 and 2015, while after 2016, due to regulation in internet finance and the transformation of P2P platforms, the space for the development of inclusive finance has been restricted and the development rate has been declining to 5.5% in 2020. Whether the development of inclusive finance is effective in promoting residents' investment plays a very important role in measuring the effectiveness of policy implementation of inclusive finance in China.

Since the State Council issued the “Plan for Promoting the Development of Inclusive Finance (2016–2020)” in 2015, China's inclusive finance has developed rapidly. Inclusive finance has expanded the coverage of financial services, improved residents' financial literacy, and raised their household income levels; at the same time, it has optimized the financial services system, enriched financial services, and improved the functions of the financial market (He and Song, 2020). Inclusive finance can improve the accumulation of household wealth, and make poor households have the ability to use finance to escape poverty (Li, 2018). In China's efforts to achieve common prosperity, the development of inclusive finance can provide a strong internal impetus, and it is also a real need and a viable option for achieving common prosperity (Zhang, 2021).

The literature has been studied mainly in terms of the impact of financial inclusion on economic growth, sustainable development, and bank regulation, mainly arguing that the development of financial inclusion will promote economic growth (Liu et al., 2021; Shen et al., 2021; Chuc et al., 2022; Younas et al., 2022) and sustainable economic development (Zaidi et al., 2021; Essel-Gaisey and Chiang, 2022; Ozturk and Ullah, 2022; Pang et al., 2022; Tay et al., 2022; Wang et al., 2022), promote industry capital formation (Cama and Emara, 2022), and influence the level of risk-taking by banks (Banna et al., 2021; Feghali et al., 2021; Marcelin et al., 2022b), regulation (Besong et al., 2022) and equity structure (Kebede et al., 2021; Marcelin et al., 2022a), finding that financial inclusion can promote financial stability (Banna et al., 2022; Malik et al., 2022; Wang and Luo, 2022), reduce poverty (Aracil et al., 2021) and improve energy efficiency and reduce carbon emissions (Dogan et al., 2021; Chen H. et al., 2022; Khan et al., 2022; Shahbaz et al., 2022). While the improvement of the institutional environment, implementation of incentives (Singh and Ghosh, 2021; Nepal and Neupane, 2022) will contribute to the development of inclusive finance (Emara and El Said, 2021; Sawadogo and Semedo, 2021; Lee et al., 2022), and the impact of other factors on financial inclusion is also analyzed (Amponsah et al., 2021; Chu et al., 2021; Geng and He, 2021; Ghosh, 2021; Chen Y. et al., 2022; Dar and Sahu, 2022; de Jong et al., 2022; Gyasi et al., 2022), contributing to an in-depth study of financial inclusion.

Among the studies on happiness, there are two main areas of research, one is the study of investor happiness and the other is the study of resident happiness. The literature on the former focuses on the impact of investors' emotions on investment (Merkle et al., 2015; Bouri et al., 2022), while studies on the happiness of residents analyze the impact of various factors from a micro perspective (Mookerjee and Beron, 2005; Zhang et al., 2017; Ngamaba et al., 2020). Among them, some scholars believe that financial inclusion can increase the happiness of the population (Abdul and Asutay, 2022) and reduce poverty levels (Koomson and Danquah, 2021), while Tsui (2014) found that people's happiness is not only related to absolute income, but also to the average income and expected income found in the society.

Financial inclusion promotes the accumulation of household wealth and also affects residents' happiness through financial behavior and personal wealth. Shen et al. (2022) showed that digital finance not only promotes more residents to invest in financial assets but also increases the proportion of households investing in financial assets, especially among urban households, high-income households, and households in the eastern region, but on the other hand, financial inclusion also increases the risk of households falling into debt crisis (Yue et al., 2022). Some research has been conducted by scholars on the impact of investment on happiness. Residents' happiness is a comprehensive indicator of the continuity of residents' subjective attitudes toward life (He et al., 2020), and scholars have conducted in-depth analyses of it. There are many studies on the happiness of the residents (Delis and Mylonidis, 2015; Bouri et al., 2022), and some studies believe that financial investment behavior will lead to a decline in residents' happiness (Hu et al., 2019; Jiang, 2019), and others believe that investors' long-term overall investment returns have a significant positive effect on investment happiness (Yang et al., 2011). Kanungo and Gupta (2021) through a study of the digitization of the Indian banking sector, found that digitization has increased the value of banks but has failed to provide the financing needed by low-income groups, and therefore, the digitization of the Indian banking sector has not achieved the goal of financial inclusion.

Abdul and Asutay (2022) found that financial inclusion through the use of pawnshops in Malaysia between 2010 and 2016 had a significant increase in personal and social happiness. Liu et al. (2021) studied provincial panel data in China from 2011 to 2019 and found that the development of digital inclusive finance contributes significantly to economic growth and that promoting the development of SMEs and stimulating consumption are two important channels through which digital inclusive finance development affects economic growth. It can be seen that the digitalization and development of inclusive finance have different impacts on societies in different countries due to their different national conditions, and the findings on residents' happiness are not consistent. Therefore, we should try to study how inclusive finance promotes the accumulation of household wealth and whether it affects residents' happiness in China, it is of great practical importance to develop inclusive finance in China, and promote China's progress toward a well-off society, and achieve common prosperity.

In view of this, the paper examines the changes in residents' investment participation and the impact on their happiness under the development of inclusive finance in various provinces in China. The results will help to understand the policy significance of inclusive finance and improve the effectiveness of the policy. The paper also concludes with a corresponding countermeasure to promote the implementation of inclusive finance policies in China and help to achieve common prosperity.

The possible innovations of this paper are: (1) The long time span and the large sample size of the data enable a full and in-depth analysis of the role of inclusive finance and the mechanism of its impact on residents' happiness. (2) A regional and yearly heterogeneity analysis has been conducted to dissect the effectiveness of policies in different regions and years, which has a certain reference value for the implementation of inclusive financial policies in China, and it also has some significance for improving the efficiency of inclusive financial development.

## Data sources, selection of variables, and model assumptions

### Data sources

In 2013, China established the “Development of inclusive finance” as a national strategy. In order to study the impact of the policy, this paper analyses data from 2013 onwards. The micro data is based on 2013, 2015, and 2017 surveys published by the China General Social Survey (CGSS) and the Digital Financial Inclusion Index of China (PKU_DFIIC) published by Peking University. For better analysis, this paper collated the initial data according to the following methods: (1) Samples with missing information and incorrect information were excluded. (2) Due to the need to analyze residents' investment behavior, in order to avoid the influence of employment, retirement, and government subsidies on investment behavior, reference was made to Hu et al.'s (2019) method, the sample age was set to be between 18 and 65 years old, and the lowest 5% of the sample in terms of household income was excluded. This paper finalized the sample data of 23,205 and shrink the tail for continuous variables in order to reduce the effect of extreme values.

The Peking University Digital Financial Inclusion Index of China (PKU_DFIIC) is used as the indicator for financial inclusion (Guo F. et al., 2020). In order to match the micro data, the data of 2013, 2015, and 2017 were used.

### Selection of variables

1. Explanatory variable: residents' investment (Investment). In this paper, we use the investment indicators from the 2013, 2015, and 2017 CGSS, which are divided into stocks, funds, bonds, derivatives, etc. If residents have an investment in each of these items, the value is 1, and if they do not have any investment, the value is 0, to indicate the extent of residents' participation in investment.
2. Explanatory variables: Inclusive finance index. This paper uses the China Digital Inclusive Finance Index published by the Digital Finance Research Center of Peking University for provinces and the inclusive finance index is standardized to the interval [0, 10] in order to better analyze their relationship.
3. Control variables: Based on the experience of previous scholars (Tay et al., 2022), this paper controls for various indicators that may affect individual investment, including information on an individual's age, income, number of properties, marital status, and so on. The age of the individual is the logarithm of the current year of the survey minus the year of birth plus one, with two-sided tailing; income is the logarithm of the total income of the whole family in the previous year; the number of properties is the number of properties owned by the whole family; marital status, education, work, and health status are the same as those taken from the CGSS questionnaire; the indicator of happiness is derived from the questionnaire “In general, are you satisfied with your living conditions?,” adjusted according to the CGSS questionnaire, As the questionnaire on happiness reads “In general, do you feel that you are happy in your life?,” the answer options are: very unhappy with a value of 1, rather unhappy with a value of 2, not happy with a value of 3 and not happy with a value of 3 and comparative happy is assigned a value of 4 and very happy is assigned a value of 5. Higher the happiness, the bigger the value.

From Table 1, we can see that the mean value of the investment is 0.098, the number of people who invest is relatively small, and after analysis, we found that the proportion of investment is increasing year by year, from 8.3% in 2013 to 12% in 2017. The mean value of happiness is 3.832, and the happiness increased from 3.77 in 2013 to 3.88 in 2015, and then decreased to 3.85 in 2017, which shows that the happiness of the residents has stabilized at a high level in recent years. The Digital Financial Inclusion Index rose at a faster rate, from 162.11 in 2013 to 223.95 in 2015, reaching 281.85 in 2017.

**Table 1:** Descriptive statistics of the main variables.

**Variables**	**Description**	**Mean**	**SD**	**Min**	**Max**
Investment	Investment engagement	0.098	0.297	0	1
Happiness	Happiness	3.832	0.812	1	5
DFIIC	Digital inclusive finance	223.606	50.035	118.01	336.65
Phjr	Financial inclusion (standardization)	5.347	2.306	1	10
Stock	Investments (equities)	0.072	0.258	0	1
Fund	Investments (funds)	0.039	0.193	0	1
Bond	Investments (bonds)	0.008	0.090	0	1
Age	Logarithm of age plus 1	44.651	12.472	18	65
xb	Gender	1.518	0.500	1	2
Health	Health	3.781	1.021	1	5
Edu	Education	5.494	3.148	1	14
Job	Work	2.530	1.764	1	6
Mar	Marriage	2.968	1.084	1	7
House	Number of properties	1.123	0.892	0	96
Income	Logarithm of total revenue	76757.62	254000	5000	1.00e+07

### Model assumptions

Inclusive finance will increase the availability of financial products for residents, and reduce the cost of financing for residents, thereby increasing the total investable assets of microeconomic entities and promoting investment. The rapid development of internet financial enterprises has provided a greater number of financial products and investment opportunities with lower thresholds, so increasing the participation of residents in investment. Zhou et al. (2020) argue that financial inclusion promotes households to invest in risky assets mainly by increasing their income. In response to the previous analysis, in order to analyze the policy effects of financial inclusion to analyze whether it promotes residents' participation in financial investment, this paper proposes the following hypotheses.

H1: The implementation of financial inclusion has promoted the investment of residents.

Based on the above assumptions, the following model is proposed in this paper.


(1) $$\begin{array}{l} Investment=\beta_{0}+\beta_{1}\times Phjr+\beta_{2}\times Controls+\varepsilon \end{array}$$

Where *Investment* is the household investment participation, with 1 for one or more stocks, funds, bonds, and other investment types and 0 for none; *Phjr* is the standardized provincial financial inclusion index; Referring to the experience of previous scholars (Hu et al., 2019; Zhou et al., 2020), Controls are control variables, including personal information on residents' gender, education, work, etc. Among the control variables chosen for this paper, those that have a significant impact on investment are age, education, marriage, number of properties, and income. Higher age and education can be considered as a sign of more experience and financial knowledge, and more likely to participate in investing due to more confidence in investing. Several other control variables are also expressions of experience and income, with higher age being associated with a higher proportion of marriage, the number of properties being an indirect representation of one's capital, and income being a direct representation of capital and therefore all having a positive effect on investment. ε is the error term.

According to Yang et al. (2011), the larger the investment size as a proportion of household assets, the higher the investment happiness, but the larger the absolute size of the investment, the lower the investment happiness (Yang et al., 2011). Hu et al. (2019) indicate that financial inclusion increases the happiness of residents through the impact of investment and income. Ma et al. (2021) find that financial risk investment can significantly increase household happiness in the short term, but has no significant effect in the long term. In order to analyze in depth mechanism of the policy effect on financial inclusion, this paper proposes hypothesis (2).

H2: The implementation of financial inclusion promotes investment and affects the happiness of the residents.

Based on the above assumptions, the following model is proposed in this paper.


(2) $$\begin{array}{l} Happiness=\beta_{0}+\beta_{1}\times Phjr+\beta_{2}\times Controls+\varepsilon \end{array}$$

Where *Happiness* is an indicator of residents' happiness, taking values from 1 to 5, with larger values being happier. In this paper, we use binary variables with the range of [0,1] for anlayse, and divide the happiness index between 1 and 3 in the questionnaire into the unhappy group with a value of 0, and the happiness index between 4 and 5 into the happy group with a value of 1. The other variables are explained in the same way as in Eq. 1.

## Empirical results

### Baseline regression

As the explanatory variables in this paper are dichotomous, a probit model was used to regress (Zhou et al., 2020) and find the marginal effects of each variable with reference to the experience of previous scholars. In order to prevent the influence of other factors on investment and happiness, variables such as personal information and income are controlled.

In Table 2, column (1) reports the basic regression, column (2) controls for control variables that may affect investment, column (3) clusters at the city level, and column (4) fixes the year effect. This paper finds a positive effect of financial inclusion on investment participation, all of which pass the significance test at the 1% level.

**Table 2:** Baseline model regression results.

**Variable name**	**Investment**			
	**(1)**	**(2)**	**(3)**	**(4)**
Inclusive finance	0.0224^***^	0.00625^***^	0.00625^***^	0.0253^***^
	(0.000864)	(0.000766)	(0.00217)	(0.00667)
Control variables	N	Y	Y	Y
City fixed effects	N	N	Y	Y
Year fixed effects	N	N	N	Y
Observations	23,205	23,205	23,205	23,205

*Robust standard errors in parentheses*.

*** *p < 0.01*.

The regression results in Table 2 show that financial inclusion has a significant boost on residents' investment participation. According to the results in column (4), each unit increase in financial inclusion, with all other variables held constant, will promote a 2.53% increase in investment participation.

In order to analyze the impact of financial inclusion on investment in-depth, this paper divides the provinces into east, middle, west, and east-north regions, and analyses the data by region and year respectively, the results are shown in Table 3. It can be seen that financial inclusion has significantly increased investment participation, but from a regional perspective, the marginal effect on the east and middle regions are significant, however, the direction of the coefficients is different. The promotional effect of financial inclusion on investment in the eastern region offsets the negative effect in the central region so that financial inclusion, in general, promotes the participation of residents in investment. When analyzed by year, it can be seen that the development of financial inclusion significantly increased residents' participation in investment in 2013, 2015, and 2017, and the coefficient shows that the impact of China's financial inclusion policy on investment is gradually strengthening and may have a more pronounced effect in the future.

**Table 3:** Heterogeneity regression results for financial inclusion on investment.

**Variable name**	**Investment**							
	**(1)** **All**	**(2)** **East**	**(3)** **Middle**	**(4)** **West**	**(5)** **East-north**	**(6)** **2013**	**(7)** **2015**	**(8)** **2017**
Inclusive Finance	0.0253^***^	0.0325^*^	−0.0223^**^	−0.00280	0.00417	0.0208^**^	0.0233^***^	0.0312^***^
	(0.00667)	(0.0183)	(0.00892)	(0.00567)	(0.00694)	(0.00992)	(0.00537)	(0.00486)
Control variables	Y	Y	Y	Y	Y	Y	Y	Y
City fixed effects	Y	Y	Y	Y	Y	Y	Y	Y
Year fixed effects	Y	Y	Y	Y	Y	N	N	N
Pseudo R^2^	0.2747	0.2011	0.2475	0.2090	0.2389	0.2692	0.2437	0.3030
Observations	23,205	8,960	5,523	5,593	3,129	7,776	7,274	8,155

*Robust standard errors in parentheses*.

*** *p < 0.01*;

** *p < 0.05*;

* *p < 0.1*.

### Robustness tests

To prevent bias in the model, this paper will use Ordinary Lease squares (OLS) to test whether the regression is biased, and the results are shown in Table 4. For the overall sample, the impact of financial inclusion on investment is largely consistent with the previous analysis in this paper, with the coefficients moving in the same direction, although slightly less significant but indicating that the model is still robust.

**Table 4:** Robustness test regression results.

**Variable name**	**Investment**							
	**(1)** **All**	**(2)** **East**	**(3)** **Middle**	**(4)** **West**	**(5)** **East-north**	**(6)** **2013**	**(7)** **2015**	**(8)** **2017**
Inclusive finance	0.0412^**^	0.0294	−0.0303^*^	−0.0055	0.0008	0.0374	0.0375^***^	0.0475^***^
	(3.20)	(1.45)	(−3.44)	(−0.87)	(0.14)	(1.94)	(4.41)	(4.72)
Control variables	Y	Y	Y	Y	Y	Y	Y	Y
City fixed effects	Y	Y	Y	Y	Y	Y	Y	Y
Year fixed effects	Y	Y	Y	Y	Y	N	N	N
R^2^	0.1841	0.1823	0.1089	0.0705	0.0939	0.1668	0.1540	0.2219
Observations	23,205	8,960	5,523	5,593	3,129	7,776	7,274	8,155

*Robust standard errors in parentheses*.

*** *p < 0.01*;

** *p < 0.05*;

* *p < 0.1*.

### Further analysis

According to previous research by scholars, financial inclusion raises residents' income which will have an impact on residents' happiness. This paper further analyses the impact of financial inclusion on residents' happiness, and the results are shown in Table 5.

**Table 5:** Regression results of the impact of financial inclusion on happiness.

**Variable name**	**Happiness**							
	**(1)** **All**	**(2)** **East**	**(3)** **Middle**	**(4)** **West**	**(5)** **East-north**	**(6)** **2013**	**(7)** **2015**	**(8)** **2017**
Inclusive finance	−0.0233^***^	−0.0293^**^	−0.0326	0.00210	−0.0669^**^	−0.0291^**^	−0.0305^**^	−0.0116
	(0.0078)	(0.0118)	(0.0208)	(0.0421)	(0.0274)	(0.0125)	(0.0133)	(0.0090)
Control variables	Y	Y	Y	Y	Y	Y	Y	Y
City fixed effects	Y	Y	Y	Y	Y	Y	Y	Y
Year fixed effects	Y	Y	Y	Y	Y	N	N	N
Pseudo R^2^	0.0635	0.0582	0.0802	0.0556	0.0847	0.0484	0.0706	0.0713
Observations	23,205	8,960	5,523	5,593	3,129	7,776	7,274	8,155

*Robust standard errors in parentheses*.

*** *p < 0.01*;

** *p < 0.05*.

Based on the results in Table 5, it can be seen that overall every 1 unit increase in the development of financial inclusion will decrease the residents' happiness by 2.33%. Analysis from a regional perspective shows that financial inclusion will significantly decrease residents' happiness in the east and east-north regions, while not significant in the other regions. From an annual perspective, it can be seen that financial inclusion development significantly decreased the happiness of residents in 2013 and 2015, while the effect was not significant in 2017.

In order to analyze the mechanism of the impact of inclusive financial development on residents' happiness, with reference to the study of scholars Zhou et al. (2020), this paper analyses the mediating effect of investment in the impact of inclusive financial development on happiness and finds that the impact of inclusive finance on happiness remains significant, the results of which are shown in Table 6. The Sobel-Goodman Mediation Tests were also conducted in this paper and the results showed a significant mediating effect of investment, playing a −8% mediating role in all effects.

**Table 6:** Regression results for the effect of investment on happiness.

**Variable name**	**Happiness**							
	**(1)** **All**	**(2)** **East**	**(3)** **Middle**	**(4)** **West**	**(5)** **East-north**	**(6)** **2013**	**(7)** **2015**	**(8)** **2017**
Inclusive Finance	−0.0228^***^	−0.0288^**^	−0.0337^*^	0.00238	−0.0669^**^	−0.0270^**^	−0.0306^**^	−0.0116
	(0.0754)	(0.0119)	(0.0204)	(0.0420)	(0.0274)	(0.0127)	(0.0133)	(0.00909)
Investment	−0.0141	−0.0172	−0.0453^***^	0.0306	−0.0125	−0.0540	0.00426	0.000605
	(0.0137)	(0.0180)	(0.0128)	(0.0295)	(0.0313)	(0.0348)	(0.0155)	(0.0138)
Control variables	Y	Y	Y	Y	Y	Y	Y	Y
City fixed effects	Y	Y	Y	Y	Y	Y	Y	Y
Year fixed effects	Y	Y	Y	Y	Y	N	N	N
Pseudo R^2^	0.0636	0.0584	0.0805	0.0557	0.0847	0.0493	0.0706	0.0713
Observations	23,205	8,960	5,523	5,593	3,129	7,776	7,274	8,155

*Robust standard errors in parentheses*.

*** *p < 0.01*;

** *p < 0.05*;

* *p < 0.1*.

Regionally, the mediating effect of investment is significant except in the western region. In terms of years, the results were more significant in 2013 and 2015, but the mediation effect was not significant in 2017. However, the mediation effect of investment in the impact of financial inclusion development on residents' happiness holds. However, the analysis shows that the negative impact of investment on the welfare of the population decreases year by year, with a progressively lower coefficient of −0.07, −0.009 to −0.006 in 2013, 2015, and 2017, respectively. Therefore, in the coming years, the financial inclusion policy will show a certain role in enhancing the well being of the population.

## Discussion

This paper analyses residents' investment participation and happiness by combining CGSS data for the 3 years of 2013, 2015, and 2017 with the financial inclusion development index for each province in each year. This paper finds that the development of inclusive finance has a catalytic effect on residents' investment participation, increasing the motivation of residents to buy financial products, and has a negative impact on residents' happiness, but the negative impact is decreasing year by year. The research in this paper also illustrates that the impact of the development of financial inclusion on the population may be at odds with policy goals, as may be the case in other countries, such as Erlando et al. (2020) finding that financial inclusion has led to widespread income inequality in eastern Indonesia, and Pradhan et al.'s (2021) finding that after the adoption of financial inclusion in several states in India between 1991 and 2018, there is still slow adoption of financial services by a large percentage of the population, as well as regional imbalances in development and income inequality. Also, the results of the regional heterogeneity study in this paper indicate that the impact of financial inclusion development on investment and residents' happiness in China varies significantly regionally, especially in the western region, where almost all effects are insignificant, Tay et al. (2022) are largely consistent with the findings of this paper.

The development of inclusive finance is not only for the sake of economic development, but also to improve residents' financial literacy and well-being in life. In the context of rapid economic development, it may take a longer time for China to improve the financial literacy of its residents so that financial inclusion can make a real difference.

## Conclusion

It is found that financial inclusion has a positive effect on residents' investment participation, with each 1-unit increase in financial inclusion development increasing residents' investment participation by 2.23%, holding other factors constant. The development of financial inclusion significantly boosted residents' investment participation in both the east and middle regions.

In terms of the impact on the happiness of residents, each unit's increase in financial inclusion development was associated with a 2.33% decrease in the happiness of residents, with the effect being significant in the east and east-north region. In addition, inclusive financial development would significantly reduce residents' happiness in 2013 and 2015, while the effect was not significant in 2017.

Based on the above conclusions, this paper puts forward the following suggestions: Firstly, the development of inclusive finance should continue to be vigorously pursued to increase residents' participation in investment. Inclusive finance has increased the coverage of financial services and enriched the financial business system while improving residents' financial awareness and ability and promoting higher financial returns. In the above analysis, it can be seen that among the six types of investments provided by the questionnaire, stocks account for only 7.2% even though they are relatively high, while funds and bonds account for 3.9 and 0.8%, respectively, which means around 90% of the population has no experience of investing. The low level of investment participation does not allow for the role of investment in the functioning of the economy.

Secondly, the coverage of residents' investment participation should be expanded. We should increase participation in investments, and improve the sense of happiness through investment and risk diversification (Yu and Yan, 2013). As seen above, only 7.2% of residents choose equities as their investment, and participation in other investments is even lower. This is due to the lack of financial knowledge and risk awareness of Chinese investors. The small variety of investments leads to a high concentration of risk and reduces the happiness of the residents.

Finally, in an analysis of regional heterogeneity, it is clear that the development of financial inclusion in China is more uneven and the effects vary. At the same time, the imbalance in the development of inclusive finance across regions may also lead to the slow development of inclusive finance because of the wide gap in economic development across regions. Therefore, greater policy support should be given to the development of inclusive finance, so it can improve the availability and coverage of financial services, and in turn promote a more comprehensive development of inclusive finance.

Although the main results are significant, the perspective of the analysis may not be comprehensive because the paper fails to include the rate of return on investments in the analysis framework. At the same time, the inability to collect data for consecutive years does not allow for a more detailed analysis of this mechanism in this paper. Future studies could include the rate of return on investment into the models since investments can capture happiness when they are profitable, and more emerging market countries can be studied, in order to find the different mechanisms of financial inclusion, and promote the development of financial inclusion.

## Data availability statement

Publicly available datasets were analyzed in this study. This data can be found here: the datasets CGSS for this study can be found in the Chinese National Survey Data Archive (CNSDA, http://www.cnsda.org), and the PKU_DFIIC can be found in the Institute of Digital Finance, Peking University (CNSDA, http://idf.pku.edu.cn).

## Author contributions

WS has furnished the theoretical background, designed the study, contributed to the data analysis, and wrote the first draft. QX helped with the methodology, analysis, and discussion sections. Both authors contributed to the article and approved the submitted version.

## Funding

This work was supported by Shihezi University.

## Conflict of interest

The authors declare that the research was conducted in the absence of any commercial or financial relationships that could be construed as a potential conflict of interest.

## Publisher's note

All claims expressed in this article are solely those of the authors and do not necessarily represent those of their affiliated organizations, or those of the publisher, the editors and the reviewers. Any product that may be evaluated in this article, or claim that may be made by its manufacturer, is not guaranteed or endorsed by the publisher.
